# Excess abdominal fat is associated with cutaneous allodynia in individuals with migraine: a prospective cohort study

**DOI:** 10.1186/s10194-020-1082-0

**Published:** 2020-02-04

**Authors:** Ane Mínguez-Olaondo, Iván Martínez-Valbuena, Sonia Romero, Gema Frühbeck, María Rosario Luquin, Eduardo Martínez-Vila, Pablo Irimia

**Affiliations:** 1 Department of Neurology, Clínica Universidad de Navarra, Pío XII 36, 31008 Pamplona, Spain; 2 Department of Neurology, Hospital Universitario Donostia, San Sebastián, Spain; 3 Regenerative Therapy Laboratory, Neurosciences Division, Center for Applied Medical Research (CIMA), University of Navarra, Pamplona, Spain; 4 Navarra’s Health Research Institute (IDISNA), Pamplona, Spain; 5 Endocrinology and Nutrition Department, Clínica Universidad de Navarra, Pamplona, Spain; 6 CIBER Fisiopatología de la Obesidad y Nutrición (CIBERobn), Instituto de Salud Carlos III, Madrid, Spain

**Keywords:** Cutaneous allodynia, Body fat, Body composition, BMI, Abdominal fat, Inflammation, Biomarker, Migraine chronification, Risk factors

## Abstract

**Objective:**

To investigate the specific relationship between cutaneous allodynia (CA) and the percentages of body fat (BF) and abdominal fat in migraineurs. Additionally, we compared serum levels of inflammatory biomarkers in patients with and without CA.

**Background:**

Excess abdominal fat might facilitate progressive changes in nociceptive thresholds causing central sensitization, clinically reflected as CA, which could drive migraine progression.

**Methods:**

This prospective cohort study included 80 patients with migraine (mean age 39 years, 81.2% female) and 39 non-migraine controls. We analysed each participant’s height, body weight, and body mass index (BMI). The amount and distribution of BF was also assessed by air displacement plethysmography (ADP) and ViScan, respectively. We analysed serum levels of markers of inflammation, during interictal periods.

**Results:**

We studied 52 patients with episodic migraine (EM) and 28 with chronic migraine (CM). Of the 80 patients, 53 (53.8%) had CA. Migraineurs with CA had a higher proportion of abdominal fat values than patients without CA (*p* = 0.04). The independent risk factors for CA were the use of migraine prophylaxis (OR 3.26, 95% CI [1.14 to 9.32]; *p* = 0.03), proportion of abdominal fat (OR 1.13, 95% CI [1.01 to 1.27]; *p =* 0.04), and presence of sleep disorders (OR 1.13, 95% CI [00.01 to 1.27]; *p* = 0.04). The concordance correlation coefficient between the ADP and BMI measurements was 0.51 (0.3681 to 0.6247). CA was not correlated with the mean plasma levels of inflammatory biomarkers.

**Conclusions:**

There is a relation between excess abdominal fat and CA. Abdominal obesity might contribute to the development of central sensitization in migraineurs, leading to migraine chronification.

## Background

Migraine can evolve from an episodic to a chronic form at a rate of approximately 3% per year [1], and obesity is one of the most relevant modifiable risk factors for chronification [2–4]. The mechanisms underlying the association between obesity and migraine progression are not yet completely clear, but several lines of evidence suggest that obesity may facilitate progressive changes in nociceptive thresholds, causing central sensitization and leading to migraine chronification [2, 3, 5].

Overweight and obesity, according to the World Health Organization (WHO), are defined as abnormal or excessive fat accumulation that presents a risk to health. It has been suggested that the quantity and distribution of adipose tissue are crucial to understanding the association between obesity, defined as excess body fat (BF), and migraine progression [6, 7]. Body mass index (BMI) is traditionally used to estimate BF, although, this measurement technique is unable to distinguish between lean body fat and fat mass and does not provide information about the distribution of adipose tissue [8–11]. Nevertheless, it may be possible to accurately assess BF and its distribution by air displacement plethysmography (ADP) and ViScan, respectively [7, 12]. In addition, obesity (and especially excess abdominal fat) is associated with an increase in chronic systemic inflammation [13] which may contribute to central sensitization [11, 14, 15]. Therefore, studying body composition measurements and circulating inflammatory mediators in migraineurs may provide a better understanding of the relationship between obesity and migraine [6, 11].

Enhanced central sensitization is thought to indicate an increased risk of migraine chronification [4]. Cutaneous allodynia (CA) is considered a clinical marker of central sensitization and is regarded as the perception of pain in response to non-noxious skin stimulation [12]. The presence of CA can be assessed with a simple validated questionnaire, the *Allodynia Symptom Checklist-12* or ASC-12 [16]. Several factors have been associated with an increased risk of CA in migraine patients, including female sex, high BMI values, the number of migraine days, comorbid depression and medication overuse [4, 12, 16, 17]. However, no information is available about the influence of BF, specifically increased abdominal fat, on CA.

Thus, we hypothesized that excess abdominal fat could play a role in migraine chronification by inducing central sensitization, which is clinically reflected as CA. This study aimed to investigate the relationship of BF and abdominal fat with CA in migraine patients. Additionally, we compared serum levels of inflammatory biomarkers in patients with and without CA.

## Patients and methods

### Study design

This was a prospective cohort study. We recruited a cohort of individuals with migraine and matched them to non-migraine controls, and both were examined for differences in fat related to the presence or absence of allodynia and levels of selected inflammatory biomarkers.

### Participants

We included 80 patients with migraine and 39 non-migraine controls. Patients were recruited from regular visitors to our clinic. The participants were adults aged 18 to 65 years. Patients attending our Neurology Department during this period were invited to participate in the study. All patients filled out a migraine headache diary for 1 month to establish the migraine frequency. Patients were diagnosed with episodic migraine (EM) or chronic migraine (CM) according to the International Classification of Headache Disorders (ICHD-III) criteria [18]. The exclusion criteria were as follows: (a) severe systemic disease or disease that could alter the measurements of inflammatory mediators, including: active infection, recent myocardial infarction (within 3 months), recent surgery (within 3 months), renal failure, severe liver disease, or inflammatory or neoplastic disease; (b) immunosuppressed patients requiring treatment with steroids or immunosuppressants, HIV-infected patients, people with addiction to drugs or alcohol; (c) patients who had recently started (in the prior 4 weeks) preventive migraine treatment or consumed opioids; (d) pregnancy or breastfeeding status; (e) patients with major psychiatric disorders or intellectual disability; and (f) morbidly obese patients according to BMI criteria. A complete medical record, including personal and family history, was available for all the participants, and their physical examination and neuroimaging results (when appropriate) were normal in every case. A total of 39 non-migraineur individuals matched for age, sex, and BMI, without a personal or family history of migraine headaches, and who also complied all the exclusion criteria were recruited from among the hospital personnel as a control group. None of them had chronic pain conditions. All the participants provided written informed consent to participate prior to their enrolment in the study. This work was approved by the local research ethics committee and was conducted in accordance with the Declaration of Helsinki.

After a screening visit, eligible individuals were invited to complete a structured questionnaire designed to obtain demographic and clinical data such as age, sex, type of migraine (episodic or chronic), duration of the disease (years), frequency of attacks per month, analgesic consumption in the last month (no opioids were used), use of migraine prophylaxis, and different body composition parameters (height, weight, BMI, abdominal fat by ViScan or body fat percentage). Patients completed a thorough history about the presence of depressive and anxiety symptoms, and sleep problems (insomnia, restless legs syndrome, sleep apnoea and excessive daytime sleepiness). Patients with suspicion of anxiety or depression during the clinical interview were referred to Psychiatry. The Structured Clinical Interview for Statistical Manual of Mental Disorders, Fifth Edition (DSM-5) was used to identify and characterize anxiety and depression. A diagnosis of depression, anxiety or sleep disorder was also made in patients who had received a previous medical diagnosis, used anti-depressants or anxiolytics (with indication for depression or anxiety), or used treatments for sleep disorders. In addition, the *Migraine Disability Assessment* (MIDAS) [19], *the Headache Impact Test-short form*™ (HIT-6) [20], and *the 12-item Allodynia Symptom Checklist* (ASC-12) questionnaires [16] were also completed. The protocol included blood extraction in a subset of patients and all controls. All data were fully anonymised prior to analysis for this study.

### Anthropometric and body composition measurements

All anthropometric and body composition measurements were obtained on the same day. Height was measured to the nearest 0.1 cm with a Holtain stadiometer (Holtain Ltd., Crymych, UK) and body weight was measured to the nearest 0.1 kg with a calibrated ADP electronic scale. Each participant’s BMI was calculated according to WHO recommendations [21, 22].

Visceral and abdominal adiposity were quantified with an abdominal BIA ViScan device (Tanita AB-140, Tanita Corp., Tokyo, Japan). This device measures total abdominal adiposity (percentage abdominal fat [range: 0 to 75%]), including subcutaneous abdominal and intra-abdominal adipose tissue expressed as ‘visceral fat’ (range: 1 to 59 arbitrary units [a.u.]). As stated by the manufacturer, visceral fat a.u. obtained by the ViScan multiplied by 10 correspond to intra-abdominal adipose tissue measured by computed tomography in cm^2^.

Body density was calculated by ADP (Bod-Pod®, Life Measurements, Concord, California, USA), to estimate fat-free mass and fat mass. The percentage of BF (%BF) was calculated from the body density using the Siri equation. Based on the most frequently used criteria in the literature [23], the %BF cut-off points used to define individuals who were overweight were: 20.1% to 24.9% for men and 30.1% to 34.9% for women; for obese participants it was: 25.0% or more for men and 35.0% or more for women. True obesity was determined as the proportion of abdominal fat mass, which was distinguished from the peripheral and appendicular fat mass as a measure of abdominal adiposity.

### Blood determinations

Venous blood serum samples were collected in chemisty test tubes in the morning after an overnight fast to avoid potential confounding influences resulting from hormonal rhythmicity, centrifuged at 3000 g for 15 min, and immediately frozen and stored at 80 °C. Commercially available ELISA kits were used to assess circulating levels of pro-inflammatory cytokines including interleukin (IL-6; RayBiotech, Inc., Norcross, GA), and tumour necrosis factor-alpha (TNF-α; R&D Systems, Abingdon, UK) according to the manufacturer’s instructions. The intra- and inter-assay coefficients of variation were: less than 10% and 12% for IL-6, and 5.4% and 8.3% for TNF-α respectively. All samples and calibrator controls were run in duplicate and the average values were calculated blinded to the clinical data.

### Assessment of cutaneous allodynia

The ASC-12, which includes 12 questions about the frequency of allodynia symptoms in association with headache attacks, was used to assess CA [16]. The responses were scored from 0 to 2, where: 0 = ‘never’, ‘rarely’, or ‘does not apply to me’; 1= ‘less than half the time’; and 2= ‘half the time or more’. The ASC-12 yields overall scores ranging from 0 to 24 and defines the following categories: no allodynia (0 to 2 points), mild allodynia (3 to 5 points), moderate allodynia (6 to 8 points), and severe allodynia (9 points or more). Previous validation analysis suggested that CA has three factors: thermal, mechanical static, and mechanical dynamic [16]. The thermal factor, reflects pain sensitivity to heat and cold and includes five items (shaving your face, taking a shower, resting your face or head on a pillow, exposure to heat, and exposure to cold). The mechanical static factor is composed of five items (wearing eyeglasses, wearing contact lenses, wearing earrings, wearing a necklace, and wearing tight clothing) and reflects fixed-point pressure. The mechanical dynamic factor comprises two items (combing your hair and pulling your hair back) and reflects a more dynamic pressure across an area of skin [16].

### Statistics

Before commencing the study, we determined that, assuming a migraineur/control proportion of 2:1, a standard deviation of 7.5 in both groups, a significance level of 0.05, and a power of 0.90, a sample of at least 73 patients in the migraine group and 37 patients in the control group would be required to detect a mean difference exceeding or equal to 5 abdominal fat units. To account for potential dropouts, we included 80 migraine patients and 39 non-migraine volunteers.

All statistical analyses were performed using SPSS software, version 15.0.1 (SPSS, Chicago, IL, USA), and *p*-values lower than 0.05 were considered statistically significant. The data were summarized using frequency counts and descriptive statistics, and presented as the mean ± *SD*. The ASC scores were evaluated as indicated above. The Kolmogorov–Smirnov test was used to test the normality of the distributions. The means of normally distributed independent samples were compared using two-tailed Student’s *t*-test. Nonnormally distributed data were compared with Mann–Whitney U tests. Categorical variables were compared using the Chi-square test and Fisher’s exact test, as required.

We compared the correlation and concordance of the %BF classification versus the BMI classification using Lin’s concordance coefficients (ρc). We used Lin’s coefficient of correlation instead of Pearson’s, because the latter estimates the general linear association and not the association specific to the equivalence line [24]. The ρc reproducibility was considered almost perfect (substantial) when values exceeded 0.99; values between 0.95 and 0.99 they were moderate; and those below 0.90 were poor [11].

A logistic regression model for the univariate analysis and multivariate multiple regression model for the multivariate analyses were used to test the association between CA, as the dependent variable, and different study variables. Variables were entered into the model by the backward strategy. The multivariate analysis included factors associated with success in the univariate analysis at *p* ≤ 0.1. Moreover, a supplementary analysis was performed with the pro-inflammatory cytokines and another substituting abdominal fat values for obesity and body fat values. The model was adjusted by age, MIDAS results, HIT-6 results and years with migraine. To ensure that collinearity was not a problem in the regression analysis, we the used variance inflation factor.

## Results

A total of 119 Caucasian individuals were included in our analyses: 80 migraine patients (52 with EM and 28 with CM) and 39 nonmigraine controls. The mean age of the migraineurs was 39 ± 10 years, and 81.2% were female (37 ± 8 years for EM and 41 ± 12 years for CM, 78.8% and 85.7% female, respectively). The mean age of the nonmigraine controls was 41 ± 10 years, and 79.5% were female. There was no significant difference in age, sex, BMI, or body composition between the patients and controls (Table 1).

**Table 1:** Anthropometric characteristics of migraine patients versus non-migraine controls

Variables	Non-migraine controls (*n* = 39)	Migraineurs (*n* = 80)	*p*-value ^a^
Age (years)	41 ± 10	39 ± 10	0.56
Female (%)	79.5%	81.2%	0.82 ^b^
Height (m)	1.70 ± 0.10	1.65 ± 0.09	0.84
Weight (kg)	70.0 ± 16.0	64.3 ± 11.7	0.07
BMI (kg/m^2^)	25.5 ± 4.6	23.7 ± 3.5	0.05
Body composition – ViScan abdominal fat	29.8 ± 12.2	34.4 ± 8.3	0.09
Body fat percentage	32.4 ± 10.5	31.9 ± 8.7	0.54 ^b^

*BMI* body mass index

^a^Two-tailed independent samples, Student’s *t*/Mann–Whitney U tests

^b^Chi-squared test

**Fig. 1:**
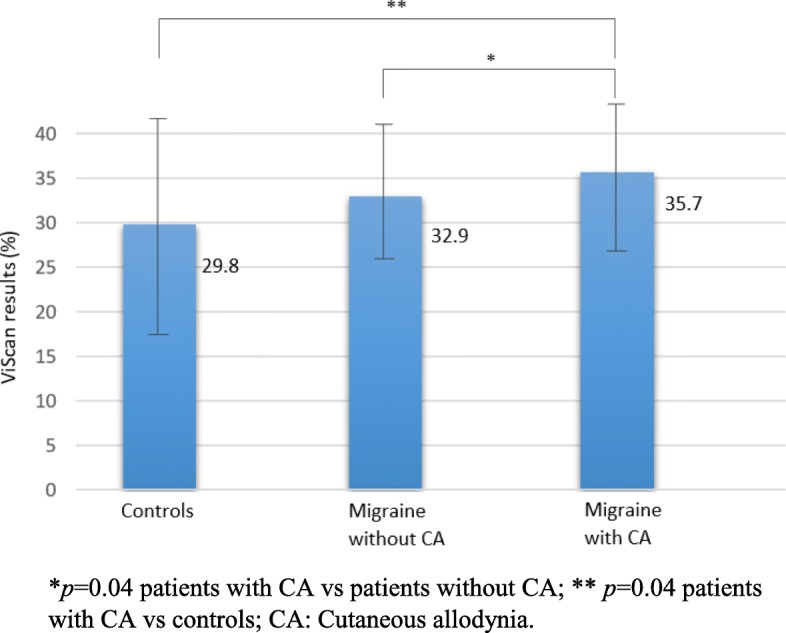
Abdominal fat in patients with or without cutaneous allodynia. *p = 0.04 patients with CA vs patients without CA; ** *p* = 0.04 patients with CA vs controls; CA: Cutaneous allodynia

In this cohort, 43 migraine patients (53.8%) had CA during their migraine attacks. Patients with CA had higher subcutaneous abdominal fat (35.7 ± 8.7), compared to patients without CA (32.9 ± 7.5, *p =* 0.04) and controls (29.8 ± 12.2, *p =* 0.04) (Fig. 1). However, there were no significant differences in subcutaneous abdominal fat between the whole sample of migraine patients and controls (*p =* 0.09). The appearance of CA in migraineurs was also associated with older age (*p =* 0.05), higher HIT-6 (62 ± 7 vs. 58 ± 9; *p =* 0.03) and MIDAS scores (54 ± 57 vs. 29 ± 40; *p =* 0.05), an increase in analgesic consumption (11 ± 8 vs. 7 ± 7; *p =* 0.03), the use of migraine prophylaxis (59.5% vs. 32.4%; *p =* 0.02), and sleep disorders (69.8% vs. 43.2%, *p =* 0.02; Table 2). Bod-Pod analysis indicated a trend towards higher %BF scores in patients with allodynia. None of the variables analysed significantly differed in relation to the type or intensity of CA. Thus, independent risk factors for the development of CA were the use of migraine prophylaxis (OR 3.26, 95% CI [1.14 to 9.32]: *p =* 0.03), abdominal fat (OR 1.13, 95% CI [1.01 to 1.27]: *p =* 0.04), and sleep disorders (OR 1.13, 95% CI [0.01 to 1.27]: *p =* 0.04).

**Table 2:** Anthropometric and clinical characteristics in migraine patients with and without cutaneous allodynia

Variables	Migraineurs with CA (*n* = 43)	Migraineurs without CA (*n* = 37)	*p*-value ^a^
Age (years)	41 ± 11	37 ± 9	0.05
Female (%)	86.5%	75.7%	0.24
Height (m)	1.64 ± 0.09	1.65 ± 0.08	0.52
Weight (kg)	64.7 ± 11.2	64.1 ± 12.4	0.79
BMI (kg/m^2^)	24.1 ± 3.9	23.4 ± 3.1	0.42
Body composition – ViScan abdominal fat	35.7 ± 8.7	32.9 ± 7.5	0.04
Body fat percentage	32.6 ± 9.4	31.1 ± 7.78	0.07
HIT-6	62.68 ± 7.19	58.47 ± 9.03	0.03
MIDAS	54.83 ± 57.83	29.03 ± 40.09	0.05
Migraine evolution (years)	9.9 ± 8.6	9.7 ± 10.3	0.03
Frequency (attacks per month)	9.49 ± 8.70	5.25 ± 5.17	0.23
1–9 days per month	23 (53.5%)	28 (75.0%)	
10–14 days per month	1 (2.3%)	1 (2.8%)	0.31 ^b^
> 15 days per month	19 (44.2%)	8 (22.2%)	
Analgesic consumption (last month)	11.32 ± 8.01	7.36 ± 7.39	0.03
Triptan consumption (last month)	4.47 ± 6.69	3.05 ± 6.51	0.464
Medication overuse	21 (48.8%)	10 (27.0%)	0.726 ^b^
Use of migraine prophylaxis	25 (59.5%)	12 (32.4%)	0.02 ^b^
Depression (%)	16.2%	10.8%	0.49 ^b^
Anxiety disorders (%)	37.2%	37.5%	0.95 ^b^
Sleep disorders (%)	69.8%	43.2%	0.02 ^b^

Anxiety and mood disorders as well as sleep disorders were self-reported

*BMI* body mass index, *CA* cutaneous allodynia, *HIT-6* 6-item Headache Impact Test, *MIDAS Migraine* Disability Assessment

^a^ Two-tailed independent samples Student’s *t*/Mann–Whitney U tests

^b^ Chi-squared test

The concordance coefficient between %BF and BMI measurements was 0.51 (0.3681 to 0.6247) with a Pearson correlation coefficient of 0.72 (0.57 to 0.82; *p* < 0.0001), while in the controls the concordance coefficient between the ADP and BMI measurements was 0.04 (− 0.1396 to 0.2204). With a sample of 52 patients in the episodic group and 28 in the CM group, this analysis had 82% statistical power to detect a difference of 5 units in abdominal fat between the two groups at a significance level of 0.05. In addition, the Spearman correlation coefficient was 0.61, indicating a moderate level of correlation between the waist-to-hip ratio and abdominal fat.

We performed blood tests on 56 of the 80 patients included, of these 29 were obese, 12 were overweight, 14 were normal weight and 1 was underweight. The mean TNF-α levels were significantly higher in patients who had migraine (1.19 vs. 0.92 pg/mL; *p* < 0.001), and IL-6 levels were almost significantly different (9.23 vs. 5.47 pg/mL; *p* = 0.05) compared to these parameters in the controls. We did not find statistically significant differences between the levels of TNF and IL-6 in patients with CA compared to non-CA patients or controls.

## Discussion

In the present study, increased subcutaneous abdominal fat was independently associated with CA. This novel finding suggests that excess abdominal fat might contribute to the development of central sensitization in migraineurs. Published research about the relationship between CA and total body adiposity or abdominal adiposity is currently limited. An association between high BMI and allodynia has been suggested [12], although this relationship was not confirmed in a subsequent study [6]. This discrepancy might reflect the dependence of CA on excess abdominal BF because the use of BMI for diagnosing obesity may be inaccurate. In this work, several migraine patients with a normal BMI actually had excess BF or abdominal fat and BMI underestimated obesity compared to ADP. Thus, our findings reinforce the notion that focusing on body composition rather than BMI is preferable when studying the association between migraine and obesity [6, 11] because, according to our results and other observations [11, 25], the use of BMI for the diagnosis of obesity might be inaccurate. The relevance of abdominal fat in the risk of migraine progression has been suggested previously [6, 26–28]. In accordance with our findings, the prevalence of migraine was higher in individuals aged 20–55 years with abdominal obesity and this association was independent of overall body obesity in women (28). Furthermore, CM has already been associated with metabolic syndrome, a cluster of common abnormalities that include abdominal obesity [29].

The mechanisms that underlie the association between increased abdominal fat and CA in migraine patients are not yet well understood. One possible explanation is that excess abdominal BF, which is metabolically more active, may promote chronic low-grade systemic inflammation [11]. Thus, the release of different inflammatory mediators might decrease the threshold for the onset of a migraine attack and contribute to central sensitization [14, 30–33]. We found that the blood serum levels of only TNF-α, but not IL-6, were significantly higher in migraine patients than in the controls. These findings may suggest that the increase in BF can intensify the neurovascular inflammatory response in migraine, promoting the sensitization of central neurons with an increased risk of progression, as suggested by Di Renzo L, et al. [11]. Thus, in addition to inflammation, dysfunction in the orexin pathways and the release of neuropeptides associated with pain transmission, (such as calcitonin gen-related peptide-CGRP-) might be involved in the development of central sensitization and migraine progression [34].

We also found that CA was associated both with the use of preventive medications and disrupted sleep, but not with medication overuse. The use of migraine prophylaxis probably reflects the fact that these patients had more frequent migraine attacks and explains this association. Although drug-induced weight gain is a side effect associated with some of the medications prescribed for migraine prophylaxis, in this cohort, patients were treated with different preventive drugs, including topiramate which is associated with weight loss. Taken together, our findings, in concordance with previous observations [27], do not suggest that the use of migraine prophylaxis influences the distribution of adipose tissue. Indeed, our data agree with previous publications in which headache frequency [4, 12, 35] and poorer sleep quality [36] were both associated with CA in patients who had migraines. Overuse of antimigraine medications may induce central sensitization [17]. In our sample, the presence of medication overuse was more frequent in patients with CA, but the difference did not achieve statistical significance (Table 2).

CA is also more frequent in headache patients with major depression and in different populations of migraineurs, depression was independently associated with CA [12, 37–39]. In our cohort, depression was more frequent in patients with CA, yet the difference did not achieve statistical significance.

Our findings may have clinical implications. Excess abdominal fat, even in patients with a normal BMI, is associated with a higher risk of central sensitization, supporting the recommendation of fat loss in such individuals. Epidemiological research shows a link between obesity and the risk of migraine [40] or migraine progression [2, 3, 41]. Different studies have demonstrated a decrease in migraine frequency after weight loss, either by dietary modifications [42, 43] or bariatric surgery [44, 45]. As such, **weight loss** is generally recommended for obese and overweight migraine patients [27, 46]. If excess abdominal fat is the main driver of migraine progression, further studies are needed to test the impact of abdominal fat reduction on migraine frequency and CA reduction.

One of the main strengths of this study is the accurate diagnosis of obesity based on detailed information of body composition to distinguish between fat free mass and fat mass and the distribution of adipose tissue. Furthermore, the individuals in this cohort received a correct migraine diagnosis according to the most recent IHS criteria, and all patients were treated by a consultant neurologist attached to a headache unit. The possible limitations of this study include the fact that the subjects were recruited from a headache clinic, excluding elderly patients, and thus the cohort may not be fully representative of migraine patients in other clinical settings. Additionally, psychiatric comorbidities and insomnia are more prevalent in patients with CM and both conditions are associated with CA. Although we directly asked our patients about their mood, anxiety symptoms and sleep problems, during the clinical interview, we did not use any formal questionnaire to diagnose depression, anxiety or the quality of sleep. In addition, we study the inflammatory biomarkers in a subgroup of patients. It is possible that larger studies may have identified differences in TNF-α and IL-6 levels between patients with and without CA. Finally, another possible limitation is that many of our patients who had migraines were receiving daily prophylactic treatments which could have influenced the levels of pro-inflammatory mediators and the appearance of CA. However, for ethical reasons, migraine prophylaxis should not be discontinued.

## Conclusions

In our cohort, we found that excess abdominal fat is associated with CA, a marker of central sensitization in patients with migraine. The distribution of adipose tissue appeared to strongly influence the association between obesity and the risk of migraine chronification. Further research into the relationship between BF distribution and the risk of migraine progression is warranted.

## Data Availability

The datasets used and/or analysed during the current study are available from the corresponding author on reasonable request.

## Abbreviations

ADP: Air displacement plethysmography
ASC: Allodynia Symptom Checklist
BF: Body fat
BMI: Body Mass Index
CA: Cutaneous allodynia
CGRP: Calcitonin gen-related peptide
CI: Confidence intervals
CM: Chronic migraine
EDQL: Today’s quality of life
EM: Episodic migraine
HIT-6: the six-item Headache Impact Text
HIV: Human immunodeficiency viruses
ICHD-III: International Classification of Headache Disorders
IL-6: Interleukin 6
MIDAS: Migraine Disability Assessment Questionnaire
OR: Odds ratio
Rc: Lin’s concordance correlation coefficients
SD: Standard deviation
TNF-α: Alfa-Tumor necrosis factor
WHO: World Health Organization
